# Enhanced Supersaturation and Oral Absorption of Sirolimus Using an Amorphous Solid Dispersion Based on Eudragit^®^ E

**DOI:** 10.3390/molecules20069496

**Published:** 2015-05-25

**Authors:** Youngseok Cho, Eun-Sol Ha, In-Hwan Baek, Min-Soo Kim, Cheong-Weon Cho, Sung-Joo Hwang

**Affiliations:** 1 College of Pharmacy, Chungnam National University, Daejeon 305-764, Korea; E-Mail: yscho@yuyu.co.kr; 2 College of Pharmacy, Pusan National University, Busan 609-735, Korea; E-Mail: edel@pusan.ac.kr; 3 College of Pharmacy, Kyungsung University, Busan 608-736, Korea; E-Mail: baek@ks.ac.kr; 4 College of Pharmacy and Yonsei Institute of Pharmaceutical Sciences, Yonsei University, Incheon 406-840, Korea

**Keywords:** sirolimus, supersaturation, bioavailability, Eudragit^®^ E, solid dispersion

## Abstract

The present study aimed to investigate the effect of Eudragit^®^ E/HCl (E-SD) on the degradation of sirolimus in simulated gastric fluid (pH 1.2) and to develop a new oral formulation of sirolimus using E-SD solid dispersions to enhance oral bioavailability. Sirolimus-loaded solid dispersions were fabricated by a spray drying process. A kinetic solubility test demonstrated that the sirolimus/E-SD/TPGS (1/8/1) solid dispersion had a maximum solubility of 196.7 μg/mL within 0.5 h that gradually decreased to 173.4 μg/mL after 12 h. According to the dissolution study, the most suitable formulation was the sirolimus/E-SD/TPGS (1/8/1) solid dispersion in simulated gastric fluid (pH 1.2), owing to enhanced stability and degree of supersaturation of E-SD and TPGS. Furthermore, pharmacokinetic studies in rats indicated that compared to the physical mixture and sirolimus/HPMC/TPGS (1/8/1) solid dispersion, the sirolimus/E-SD/TPGS (1/8/1) solid dispersion significantly improved oral absorption of sirolimus. E-SD significantly inhibited the degradation of sirolimus in a dose-dependent manner. E-SD also significantly inhibited the precipitation of sirolimus compared to hydroxypropylmethyl cellulose (HPMC). Therefore, the results from the present study suggest that the sirolimus-loaded E-SD/TPGS solid dispersion has great potential in clinical applications.

## 1. Introduction

Sirolimus (also known as rapamycin) is a macrocyclic lactone produced by *Streptomyces hygroscopicus* that inhibits interleukin (IL)-2 and other cytokine receptor-dependent signal transduction mechanisms [1]. Sirolimus is an immunosuppressive agent indicated for the prophylaxis of organ rejection in patients aged ≥ 13 years receiving renal transplants [2]. Unfortunately, sirolimus belongs to the biopharmaceutics classification system (BCS) class II drug category because of its low solubility and high permeability [3]. To improve the biological performance of sirolimus, various formulations, such as inclusion complexes with cyclodextrins, liposomal formulations, nanocrystals, and solid dispersion, have been developed [4,5,6,7,8]. Our group recently reported that of the 71 combination formulations we evaluated, the most efficient ternary solid dispersion for the enhanced bioavailability of sirolimus was hydroxypropylmethyl cellulose (HPMC)/d-α-tocopheryl polyethylene glycol 1000 succinate (TPGS) [9]. Recently, Petruševska *et al*. reported that HPMC effectively created a desired microenviroment that both maintained a supersaturated solution and prevented sirolimus precipitation [10]. In addition, it was observed that *in vitro* supersaturated dissolution data strongly correlated with *in vivo* pharmacokinetic parameters [9,10]. In fact, the oral bioavailability of sirolimus can be improved by enhancing *in vitro* supersaturation via an amorphous solid dispersion.

Eudragit^®^ E is a cationic polymer based on dimethylaminoethyl, butyl and methyl methacrylate. The Eudragit^®^ E solid dispersion is a well-established formulation system that enhances the bioavailability of poorly water-soluble active pharmaceutical ingredients (APIs) [11,12,13]. An amorphous Eudragit^®^ E solid dispersion containing poorly water-soluble APIs can be manufactured via the principle of solvent evaporation, melting, and/or solvent-mediated melting using spray drying, or the hot-melt extrusion process [14,15]. Unfortunately, Eudragit^®^ E dissolves in aqueous solutions below pH 5.0; thus, drug dissolution from the Eudragit^®^ E solid dispersion decreases at a pH above 6.0 [16]. Recently, Yoshida *et al*. developed the Eudragit^®^ E/HCl (E-SD) system, which was prepared by partial neutralization of Eudragit^®^ E using hydrochloric acid for the enhancement of supersaturation and oral absorption of poorly water-soluble APIs [17,18]. They demonstrated that E-SD forms a micelle-like structure in different conditions, similar to a polymeric surfactant [19].

The present study aimed to investigate the effect of E-SD on the degradation of sirolimus in simulated gastric fluid (pH 1.2), as well as to develop a new oral formulation of sirolimus using the E-SD solid dispersion to enhance oral bioavailability. Sirolimus-loaded solid dispersions were fabricated via the spray drying process. *In vitro* and *in vivo* characterization of the solid dispersions was conducted to focus on the effect of the E-SD solid dispersion on supersaturation and the oral absorption of sirolimus.

## 2. Results and Discussion

**Figure 1:**
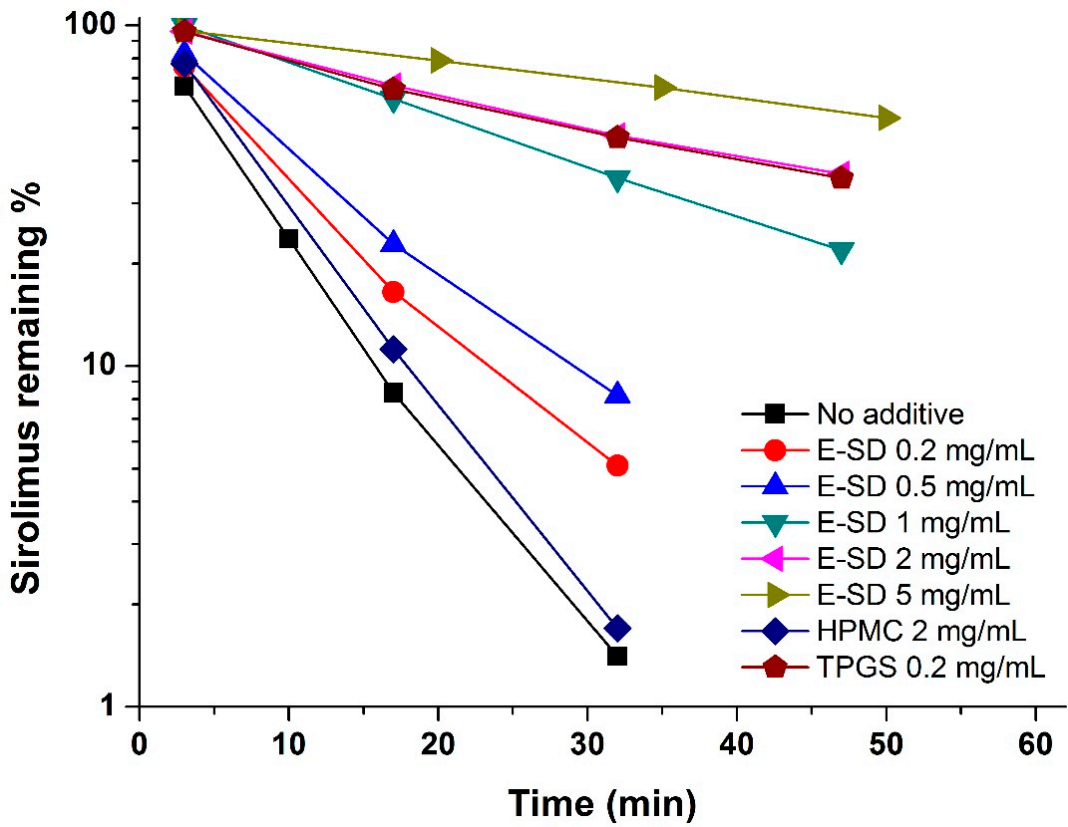
Effect of E-SD on the degradation of sirolimus in simulated gastric fluid (pH 1.2).

Sirolimus is known to be very unstable in electrolyte solutions, especially acidic ones, and is degraded via ring opening/fragmentation [20,21]. Previously, we reported that a rapid degradation of sirolimus was observed in simulated gastric fluid (pH 1.2) compared to intestinal fluid (pH 6.8), acetate buffer (pH 4.0), and distilled water [22]. Thus, the effect of E-SD on the degradation of sirolimus was further investigated in simulated gastric fluid (pH 1.2). As shown in Figure 1, the degradation of sirolimus occurred rapidly and the percent remaining was below 2% at 30 min. HPMC had no influence on degradation, while E-SD demonstrated a significant dose-dependent inhibitory effect. As expected, the pseudo-first-order degradation constant of sirolimus decreased with increasing concentrations of E-SD (Table 1). In particular, the kinetic rate of degradation of 5 mg/mL E-SD was about 10-fold lower than that of the drug alone. TPGS, a nonionic polymeric surfactant, dramatically inhibited the degradation of sirolimus by forming micelles [23]. Recently, the critical micelle concentration of E-SD was reported to be 100 μg/mL [19]. HPMC, a hydrophilic polymer, does not form micelle-like structures, while E-SD, similar to TPGS, forms micelle-like structures. Thus, the enhanced stability of sirolimus with increasing amounts of E-SD could be attributed to the formation of micelle-like structures.

To investigate the effect of the sirolimus/E-SD ratio on the improvement of sirolimus super-saturation, E-SD solid dispersions were fabricated via the spray drying method. For comparison, sirolimus-loaded HPMC and HPMC/TPGS solid dispersions were also prepared.

**Table 1:** Effect of E-SD on the degradation of sirolimus in simulated gastric fluid (pH 1.2).

Composition	*k_obs_* (min^−1^)	*t*_1/2_ (min)
No additives	0.1322 ± 0.0011	5.24 ± 0.04
E-SD 0.2 mg/mL	0.0925 ± 0.0013	7.49 ± 0.10
E-SD 0.5 mg/mL	0.0794 ± 0.0006	8.73 ± 0.07
E-SD 1 mg/mL	0.0345 ± 0.0005	20.07 ± 0.29
E-SD 2 mg/mL	0.0219 ± 0.0004	31.65 ± 0.58
E-SD 5 mg/mL	0.0125 ± 0.0005	55.64 ± 2.02
HPMC 2 mg/mL	0.1319 ± 0.0020	5.25 ± 0.08
TPGS 0.2 mg/mL	0.0225 ± 0.0009	30.83 ± 1.16

The pseudo-first-order rate constants (*k_obs_*) and half-life (*t*_1/2_) were calculated according to the equations ln[*C*_t_] = ln[*C*_0_] − *k_obs_*·*t* and *t*_1/2_ = 0.693/*k_obs_*, respectively. [*C*_0_] is the initial concentration of sirolimus and [*C*_t_] is the percentage remaining at time *t*, which allowed for the calculation of *k_obs_* as the slopes of the lines obtained by linear regression analysis. Data are expressed as the mean ± standard deviation (*n* = 3).

**Figure 2:**
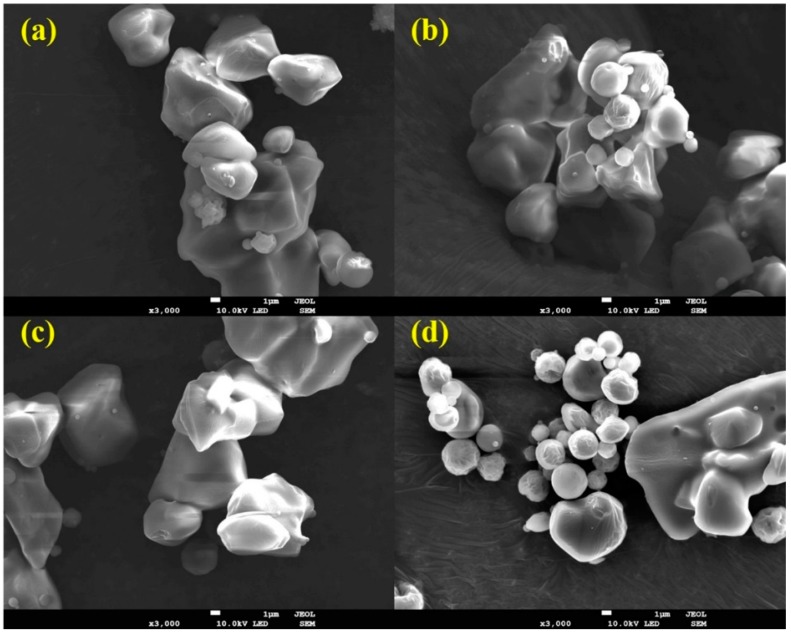
Scanning electron micrographs of sirolimus-loaded solid dispersions. (**a**) Sirolimus/E-SD solid dispersion (1/9); (**b**) Sirolimus/E-SD/TPGS solid dispersion (1/8/1); (**c**) Sirolimus/E-SD/HPMC solid dispersion (1/8/1); (**d**) Sirolimus/HPMC/TPGS solid dispersion (1/8/1). Total magnification of 3000× with a scale bar corresponding to 1 μm.

As shown in Figure 2 and Table 2, all solid dispersions had irregular-shaped particles with similar mean particle size (6–8 μm). There were no significant size differences between the solid dispersions (*p* > 0.05). The encapsulation efficiency in each solid dispersion was almost equal to that of theoretical values, as shown by HPLC analysis, indicating that sirolimus was not degraded during the spray drying process.

**Table 2:** Formulation, encapsulation efficiency, and particle size of sirolimus-loaded solid dispersions.

Formulation (Weight)	Encapsulation Efficiency (%)	Mean Particle Size (μm)
Sirolimus/E-SD = 1/1	97.9 ± 2.1	6.72 ± 1.82 (1.89) ^#^
Sirolimus/E-SD = 1/2	98.1 ± 1.8	7.43 ± 1.99 (1.90)
Sirolimus/E-SD = 1/9	96.7 ± 2.5	7.21 ± 1.79 (1.95)
Sirolimus/E-SD/TPGS = 1/8/1	98.2 ± 1.5	7.56 ± 1.95 (2.10)
Sirolimus/E-SD/HPMC = 1/8/1	96.9 ± 1.8	7.89 ± 2.03 (2.02)
Sirolimus/HPMC = 1/9	99.6 ± 1.2	6.92 ± 1.88 (1.92)
Sirolimus/HPMC/TPGS = 1/8/1	98.9 ± 1.5	6.43 ± 1.71 (2.15)

The encapsulation efficiency (%) = weight of loaded drug/weight of the feeding drug × 100. The particle size of the respective gelatin microparticle-containing self-microemulsifying formulation in the solid state was measured using a HELOS laser diffraction analyzer. ^#^ SPAN = (*d*_90%_ − *d*_10%_)/*d*_50%_, where *d*_10%_, *d*_50%_, and *d*_90%_ are the diameter sizes and the given percentage value is the percentage of the particles smaller than that size.

**Figure 3:**
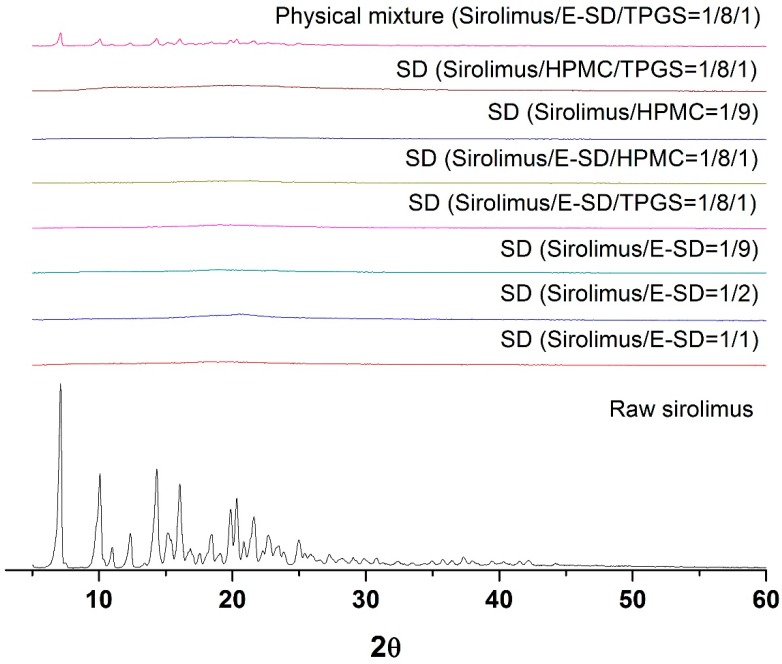
Powder X-ray diffraction patterns of sirolimus-loaded solid dispersions.

As depicted in Figure 3, the crystalline state of sirolimus within the solid dispersion was determined by powder X-ray diffractometer (PXRD, Bruker AXS GmbH, Karlsruhe, Germany). Raw sirolimus had the characteristic diffraction patterns at 2θ values of 7.2°, 9.9°, 14.5°, 16.2°, 20.0°, and 20.4°. However, the characteristic diffraction patterns of crystalline sirolimus were not observed in the PXRD patterns of all sirolimus-loaded solid dispersions, respectively. This indicates that the crystallinity of sirolimus significantly decreased, and that sirolimus exists in an amorphous form within solid dispersions.

**Figure 4:**
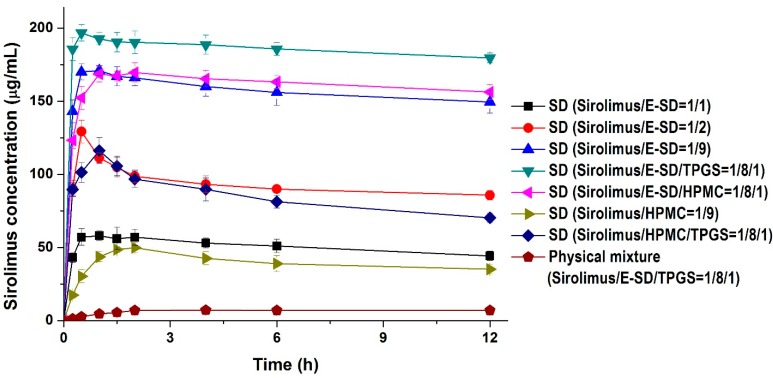
Kinetic solubility profiles of sirolimus-loaded solid dispersions. Data are expressed as the mean ± standard deviation (*n* = 3).

**Figure 5:**
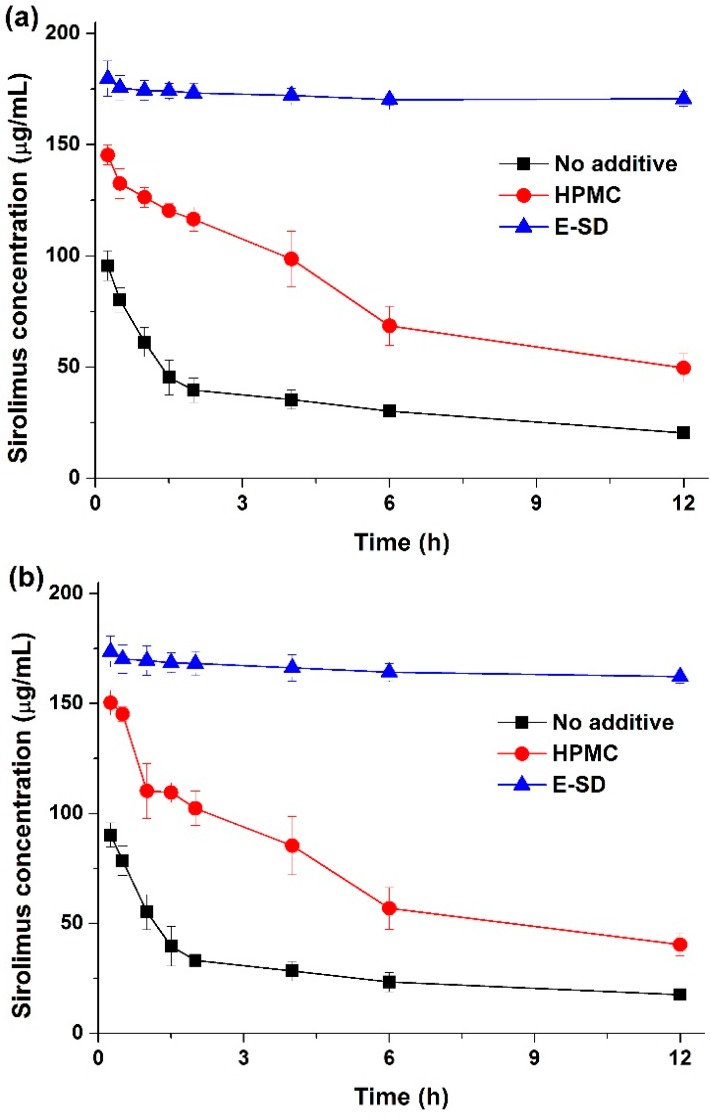
Effect of E-SD and HPMC on the recrystallization of sirolimus in two different media. Water (**a**) intestinal fluid (pH 6.8) (**b**). Data are expressed as the mean ± standard deviation (*n* = 3).

Kinetic solubility tests were performed in distilled water to investigate degree of supersaturation of sirolimus-loaded solid dispersions. As shown in Figure 4, the maximum solubility (supersaturation concentration) of sirolimus in the solid dispersions was 49.8–196.7 μg/mL, and the concentration at 12 h was 35.1–173.4 μg/mL. In particular, the maximum solubility of sirolimus dramatically increased with increasing amounts of E-SD in the solid dispersions. For example, the sirolimus/E-SD (1/9) solid dispersion showed a maximum solubility of 170 μg/mL within 0.5 h, whereas the sirolimus/HPMC (1/9) solid dispersion had a solubility of 49.8 μg/mL at 2 h, which decreased to 35.1 μg/mL after 12 h. Compared with HPMC, E-SD demonstrated higher supersaturation and prolonged superstation of sirolimus. These results suggest that E-SD might inhibit drug precipitation from the supersaturated state. Therefore, the effect of E-SD and HPMC on the recrystallization of a supersaturated sirolimus solution was investigated by the addition of a sirolimus solution in DMSO into water or simulated intestinal fluid (pH 6.8). As shown in Figure 5, sirolimus gradually precipitated in both water and simulated intestinal fluid (pH 8) without E-SD and HPMC; sirolimus is practically insoluble in water (2.6 μg/mL) and contains functional groups that are ionizable in the pH range 1–10 [24]. However, precipitation was significantly inhibited by E-SD as E-SD maintained the concentration of sirolimus above 160 μg/mL for at least 12 h. In addition, E-SD exhibited a superior inhibitory effect when compared to HPMC. The inhibition of precipitation by E-SD might be attributed to the inhibition of nucleation and/or crystal growth in a highly supersaturated state by blocking the active surface and providing steric stabilization by acting like a surfactant [25,26,27,28]. It may also be owing to the specific interactions between sirolimus and E-SD, such as hydrophobic interactions or hydrogen bonding [29,30,31]. Recently, Hiashi *et al* reported that hydrophobic and hydrophilic interactions between mefenamic acid and Eudragit EPO molecules play a key role in the formation of a stable supersaturated solution by using high-resolution magic-angle spinning NMR measurements [32]. However, this hypothesis requires further study.

The addition of TPGS to sirolimus/E-SD solid dispersions further increased supersaturation. The sirolimus/E-SD/TPGS (1/8/1) solid dispersion demonstrated a maximum solubility of 196.7 μg/mL within 0.5 h that gradually decreased to 173.4 μg/mL after 12 h. The solubility of the sirolimus/E-SD/TPGS (1/8/1) solid dispersion was higher than that of the sirolimus/HPMC/TPGS (1/8/1) solid dispersion at all timepoints. Furthermore, the sirolimus/E-SD/TPGS (1/8/1) solid dispersion showed a maximum supersaturation that was approximately 28-fold greater than the concentration of its physical mixture.

**Figure 6:**
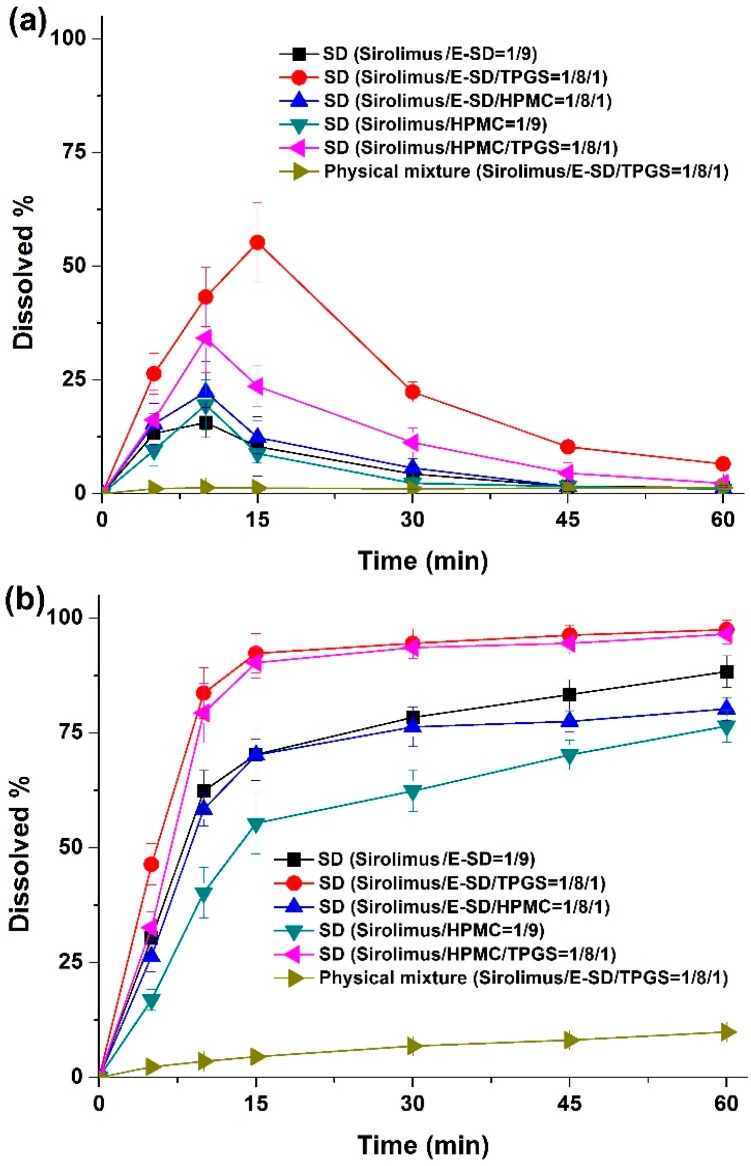
Dissolution profiles of sirolimus-loaded solid dispersions in media with different pHs. Simulated gastric fluid (pH 1.2) containing 0.05% w/v SLS (**a**); intestinal fluid (pH 6.8) containing 0.05% w/v SLS (**b**). Data are expressed as the mean ± standard deviation (*n* = 4).

Dissolution profiles were obtained for sirolimus-loaded solid dispersions in simulated gastric fluid (pH 1.2) containing 0.05% w/v sodium lauryl sulfate (SLS), and simulated intestinal fluid (pH 6.8) containing 0.05% w/v SLS as dissolution medium. As shown in Figure 6, rapid dissolution profiles were obtained for the solid dispersions in simulated intestinal fluid (pH 6.8), while characteristic convex curves were observed for dispersions in the simulated gastric fluid (pH 1.2) owing to the degradation of sirolimus. The dissolution from solid dispersions was dramatically higher than that from the physical mixture. Of the tested samples, the most suitable formulation was the sirolimus/E-SD/TPGS (1/8/1) solid dispersion in simulated gastric fluid (pH 1.2) owing to the enhanced stability and supersaturation by E-SD and TPGS. In simulated intestinal fluid (pH 6.8), approximately 90% of the drug dissolved from the sirolimus/E-SD/TPGS (1/8/1) solid dispersion, while only 10% of the drug dissolved from the physical mixture at 30 min. The dissolution profile of the sirolimus/HPMC/TPGS (1/8/1) solid dispersion was similar to the sirolimus/E-SD/TPGS (1/8/1) solid dispersion in simulated intestinal fluid (pH 6.8).

**Figure 7:**
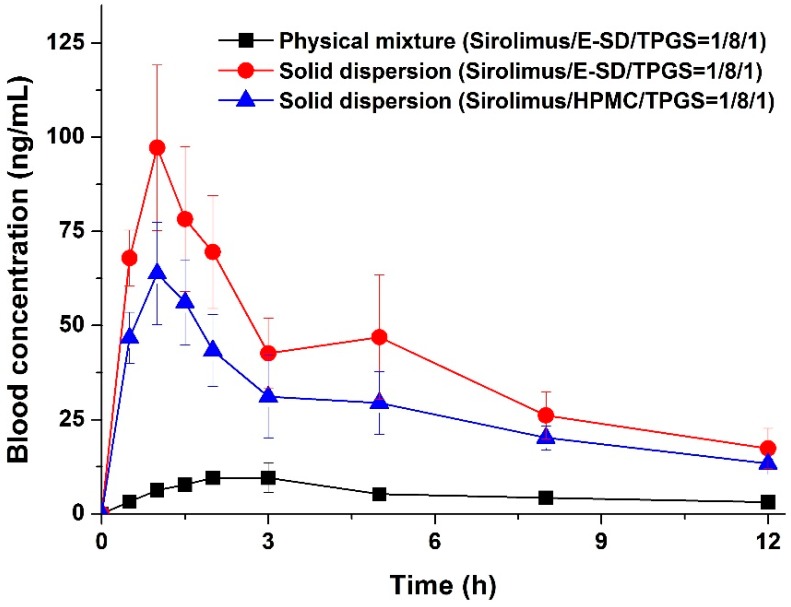
Blood concentration-time profile of sirolimus in rats after oral administration of sirolimus-loaded solid dispersions and the physical mixture. Data are expressed as the mean ± standard deviation (*n* = 5).

The enhanced supersaturation and dissolution property of sirolimus in an amorphous solid dispersion based on E-SD could be directly related to the enhanced oral bioavailability of sirolimus. Thus, we investigated the oral bioavailability of sirolimus-loaded E-SD solid dispersions in SD rats. Figure 7 shows the blood concentration-time profile of sirolimus after oral administration of both solid dispersions and the physical mixture. Pharmacokinetic parameters (AUC_0→12 h_, C_max_, and T_max_) are presented in Table 3. As shown in Figure 7, the blood concentration of the sirolimus/E-SD/TPGS (1/8/1) solid dispersion with the rapid drug absorption rate was dramatically higher than that of the physical mixture at all timepoints. The AUC_0→12 h_, C_max_, and T_max_ were 459.5 ± 93.6 ng·h/mL, 97.4 ± 19.2 ng/mL, and 1.2 ± 0.3 h, respectively. The oral absorption of the E-SD/TPGS (8/1) solid dispersion was significantly higher than that of the physical mixture and HPMC/TPGS (8/1) solid dispersion, with approximately 7.2- and 1.4-fold increases in AUC_0→12 h_, respectively (Table 3). The bioavailability of the sirolimus/E-SD/TPGS (1/8/1) solid dispersion was markedly higher than that of the physical mixture and sirolimus/HPMC/TPGS (1/8/1) solid dispersion, due to higher supersaturation and supersaturated concentration over an increased period of time, as well as enhanced stability in simulated gastric fluid (pH 1.2) [33]. These results suggest that the supersaturation and oral absorption of sirolimus were significantly increased by the amorphous E-SD/TPGS solid dispersion.

**Table 3:** Pharmacokinetic parameters of sirolimus-loaded solid dispersions in rats.

Formulation	AUC_0→12 h_ (ng·h/mL)	C_max_ (ng/mL)	T_max_ (h)
Physical mixture (sirolimus/E-SD/TPGS = 1/8/1)	64.0 ± 11.8	9.6 ± 2.6	3.8 ± 1.1
Solid dispersion (sirolimus/E-SD/TPGS = 1/8/1)	459.5 ± 93.6 ^a,b^	97.4 ± 19.2 ^a,b^	1.2 ± 0.3
Solid dispersion (sirolimus/HPMC/TPGS = 1/8/1)	325.5 ± 46.4 ^a^	61.0 ± 14.2 ^a^	1.6 ± 0.5

^a^ *p* < 0.05 *vs.* physical mixture; ^b^ *p* < 0.05 *vs.* sirolimus/HPMC/TPGS. Data are expressed as the mean ± standard deviation (*n* = 5).

## 3. Experimental Section

### 3.1. Materials

Sirolimus (purity 99.4%) and everolimus (an internal standard for LC-MC analysis, purity 95.9%) were purchased from Beijing Everbright Science & Trading Co. (Beijing, China) and Sigma-Aldrich (St. Louis, MO, USA), respectively. Eudragit^®^ E (poly(butylmethacrylateco-(2-dimethylaminoethyl)methacrylate-co-methyl methacrylate) was provided by BASF Co. Ltd. (Ludwigshafen, Germany). Hydroxypropyl-methyl cellulose (HPMC 2910) and D-α-tocopheryl polyethylene glycol 1000 succinate (TPGS) were purchased from Shin-Etsu Chemical Co Ltd (Tokyo, Japan) and Eastman Co. (Kingsport, TN, USA), respectively. Acetonitrile, dimethyl sulfoxide (DMSO) and methanol were of high-performance liquid chromatography (HPLC) grade. All other chemicals were of reagent grade.

### 3.2. Preparation of Eudragit^®^ E/HCl (E-SD) Using Spray Drying Method

Eudragit^®^ E/HCl (E-SD) was prepared by a spray drying process as previously reported [17,18]. Eudragit^®^ E (100 g) was dispersed into a mixture solution consisting of 10% (w/v) HCl (73.5 g) and 526.5 g of water; the components were mixed by mechanical stirring until a clear solution was obtained. This solution was spray dried using a B-191 mini spray dryer (Buchi, Flawil, Switzerland) under the following conditions: inlet temperature, 130–145 °C; outlet temperature, 75–85 °C; feed rate, 2–10 mL/min; and atomization air pressure, 5 kPa. Eudragit^®^ E/HCl (E-SD) was obtained as a powder.

### 3.3. Effect of E-SD, HPMC, and TPGS on the Degradation of Sirolimus in Solution

The effect of E-SD, HPMC, and TPGS on the degradation of sirolimus in solution was investigated. Sirolimus was dissolved in DMSO at a concentration of 1 mg/mL. A 100-μL aliquot of the solution was added to 50 mL simulated gastric fluid (pH 1.2, without pepsin) containing different concentrations of E-SD, HPMC, and TPGS at 37 °C in the dark. Samples were removed at various time intervals, and the concentration of sirolimus was determined by HPLC. HPLC analysis of all samples was performed with a Waters HPLC system (Waters, Milford, MA, USA) equipped with a Waters 600 controller pump, and Waters 486 tunable absorbance detector. The mobile phase was methanol/water (84:16, v/v) at a flow rate of 1.0 mL/min. All chromatographic analysis was performed on a C18 analytical column (Luna C18 (2), 5 μm, 4.6 mm × 250 mm, Phenomenex, Torrance, CA, USA) at 60 °C. Detection was performed at a wavelength of 278 nm.

### 3.4. Effect of E-SD and HPMC on the Recrystallization of Sirolimus in a Supersaturated Solution

To investigate the effect of E-SD and HPMC on the recrystallization of a supersaturated sirolimus solution, sirolimus was dissolved in DMSO at a concentration of 30 mg/mL. A 2-mL aliquot of the sirolimus solution was added to 300 mL of water or simulated intestinal fluid (pH 6.8) with/without 600 mg of an additive (E-SD or HPMC), and agitated with an USP rotating paddle apparatus (Electrolab, Mumbai, India) at 37 °C and 50 rpm. Samples (2 mL) were removed at various time intervals and filtered using a 0.45 μm glass fiber syringe filter. The filtered samples were diluted with methanol, and the concentration of sirolimus was determined by HPLC. 

### 3.5. Preparation of Sirolimus-Loaded Solid Dispersions

Sirolimus-loaded solid dispersions were fabricated via the spray drying method using a Buchi B-191 nozzle type mini spray dryer. Sirolimus was dissolved in a mixture of ethanol and methylene chloride (55:45, w/w) containing E-SD, HPMC, and/or TPGS at a concentration of 3% w/w of solute. The detailed composition of sirolimus-loaded solid dispersions are described in Table 2. The resulting solution was delivered to the inner line of the nozzle at a flow rate of 3–6 mL/min using a peristaltic pump, and solidified under the following conditions: inlet temperature, 65–80 °C; outlet temperature, 45–55 °C; and atomization air pressure, 5 kPa. For comparison, the physical mixture was also prepared by simply mixing sirolimus with E-SD, HPMC, and/or TPGS using a spatula in a glass vial.

### 3.6. Characterization of Sirolimus-Loaded Solid Dispersions

The sirolimus content in solid dispersions was determined by HPLC analysis. About 20 mg of each solid dispersion was dissolved in 50 mL of ethanol and methylene chloride (55:45, w/w). The morphology of sirolimus-loaded solid dispersions was observed by scanning electron microscopy (SEM; JSM-7100f, Jeol Ltd., Tokyo, Japan). The particle size and size distribution of sirolimus-loaded solid dispersions were characterized using a HELOS laser diffraction spectrometer (SYMPATEC Ltd., Clausthal-Zellerfeld, Germany). Powder X-ray diffraction patterns of sirolimus-loaded solid dispersions were determined using a D8 Advance X-ray diffraction system (Bruker AXS GmbH, Karlsruhe, Germany) with Ni-filtered Cu-Kα radiation. The samples were analyzed between 5° and 60°. Kinetic solubility profiles of the sirolimus-loaded solid dispersions were obtained in a water-jacketed vessel linked to a temperature-controlled water bath held at 37 °C. The solid dispersions (containing 40 mg sirolimus) were combined with 200 mL water and agitated constantly at 100 rpm. At pre-determined intervals, samples from various time points were filtered using a 0.45-μm glass fiber syringe filter followed by dilution with methanol, and the amount of drug dissolved in each sample was determined by HPLC. Dissolution profiles of sirolimus from solid dispersions were determined using an USP rotating paddle apparatus (Electrolab) at 37 °C and 100 rpm in 500 mL simulated gastric fluid (pH 1.2, without pepsin) containing 0.05% w/v SLS, and simulated intestinal fluid (pH 6.8) containing 0.05% w/v SLS as dissolution medium. After samples containing 2 mg sirolimus were placed in the dissolution medium, 3 mL samples were collected for analysis at pre-determined intervals and replaced with 3 mL of fresh dissolution medium after each collection. Samples from various time points were filtered using a 0.45-μm glass fiber syringe filter followed by dilution with methanol. The amount of drug dissolved in each sample was determined by HPLC.

### 3.7. In Vivo Study in Rats

The animal study protocol was in compliance with the institutional guidelines for the care and use of laboratory animals, and was approved by the ethics committee of Chungnam National University (Daejeon, Korea). Fifteen male Sprague-Dawley (SD) rats (250 ± 10 g, Orient Bio Inc., Seongnam, Korea) were divided into three treatment groups of five rats each. Prior to the study, the rats fasted for 18 hours; each group received either the physical mixture or solid dispersions at sirolimus doses of 5 mg/kg (dose/rat weight) for each formulation by oral gavage. The physical mixture and sirolimus-loaded solid dispersions were dispersed in 1 mL water immediately prior to oral dosing. Approximately 0.45 mL blood samples were collected in heparinized tubes from the retro-orbital plexus of rats at 0.5, 1, 1.5, 2, 3, 5, 8, and 12 h after dosing. Blood concentrations of sirolimus were determined by liquid chromatography with mass spectrometry (LC-MS), according to our previously reported method [21]. Pharmacokinetic analysis of the data was carried out with WinNonlin Standard Edition software, version 5.3 (Pharsight Corp., St. Louis, MO, USA, 2009). The area under the curve (AUC_0__→__12 h_) was calculated according to the trapezoidal method. The peak blood concentration (C_max_) and the time to reach C_max_ (T_max_) of sirolimus in the plasma were taken directly from the data. Statistical analysis for the dissolution data and pharmacokinetic parameters was performed using a one-way analysis of variance (ANOVA) test followed by the Student-Newman-Keuls (SNK) and least-squares difference (LSD) tests with SPSS 21.0 software (IBM SPSS Statistics, Armonk, NY, USA, 2013).

## 4. Conclusions

In this study, E-SD significantly inhibited the degradation of sirolimus in a dose-dependent manner. In addition, E-SD exhibited a superior inhibitory effect on the precipitation of sirolimus compared to HPMC. Sirolimus-loaded solid dispersions were fabricated by the spray drying process. In both the kinetic solubility and dissolution test, the most suitable formulation was the sirolimus/E-SD/TPGS (1/8/1) solid dispersion owing to the enhanced stability and degree of supersaturation of E-SD and TPGS. Furthermore, pharmacokinetic studies in rats indicated that compared to the physical mixture and sirolimus/HPMC/TPGS (1/8/1) solid dispersion, the sirolimus/E-SD/TPGS (1/8/1) solid dispersion significantly improved the oral absorption of sirolimus. Therefore, the results from our study suggest that the sirolimus-loaded E-SD/TPGS solid dispersion has great potential in clinical applications.
